# Mechanical Properties of Orthodontic Cements and Their Behavior in Acidic Environments

**DOI:** 10.3390/ma15227904

**Published:** 2022-11-09

**Authors:** Cristina Iosif, Stanca Cuc, Doina Prodan, Marioara Moldovan, Ioan Petean, Anca Labunet, Lucian Barbu Tudoran, Iulia Clara Badea, Sorin Claudiu Man, Mîndra Eugenia Badea, Radu Chifor

**Affiliations:** 1 Department of Prosthetic Dentistry and Dental Materials, “Iuliu Hatieganu” University of Medicine and Pharmacy, 32 Clinicilor Street, 400006 Cluj-Napoca, Romania; 2 Department of Polymer Composites, Institute of Chemistry “Raluca Ripan”, University Babes-Bolyai, 30 Fantanele Street, 400294 Cluj-Napoca, Romania; 3 Faculty of Chemistry and Chemical Engineering, University Babes-Bolyai, 11 Arany János Street, 400028 Cluj-Napoca, Romania; 4 Department of Molecular Biology and Biotechnology, Electron Microscopy Laboratory, Biology and Geology Faculty, Babes-Bolyai University, 5–7 Clinicilor Str., 400006 Cluj-Napoca, Romania; 5 Electron Microscopy Integrated Laboratory, National Institute for Research and Development of Isotopic and Molecular Technologies, 65-103 Donath Street, 400293 Cluj-Napoca, Romania; 6 Dental Prevention Department, Faculty of Dental Medicine, “Iuliu Hatieganu” University of Medicine and Pharmacy, Avram Iancu 31, 400083 Cluj-Napoca, Romania; 7 Mother and Child Department, 3Rd Department of Paediatrics, “Iuliu Hatieganu” University of Medicine and Pharmacy, 2-4 Campeni Street, 400217 Cluj-Napoca, Romania

**Keywords:** orthodontic cements, acidic environment, mechanical properties, surface roughness

## Abstract

The present research is focused on three different classes of orthodontic cements: resin composites (e.g., BracePaste); resin-modified glass ionomer RMGIC (e.g., Fuji Ortho) and resin cement (e.g., Transbond). Their mechanical properties such as compressive strength, diametral tensile strength and flexural strength were correlated with the samples’ microstructures, liquid absorption, and solubility in liquid. The results show that the best compressive (100 MPa) and flexural strength (75 Mpa) was obtained by BracePaste and the best diametral tensile strength was obtained by Transbond (230 MPa). The lowestvalues were obtained by Fuji Ortho RMGIC. The elastic modulus is relatively high around 14 GPa for BracePaste, and Fuji Ortho and Transbond have only 7 GPa. The samples were also subjected to artificial saliva and tested in different acidic environments such as Coca-Cola and Red Bull. Their absorption and solubility were investigated at different times ranging from 1 day to 21 days. Fuji Ortho presents the highest liquid absorption followed by Transbond, the artificial saliva has the best absorption and Red Bull has the lowest absorption. The best resistance to the liquids was obtained by BracePaste in all environments. Coca-Cola presents values four times greater than the ones observed for artificial saliva. Solubility tests show that BracePaste is more soluble in artificial saliva, and Fuji Ortho and Transbond are more soluble in Red Bull and Coca-Cola. Scanning electron microscopy (SEM) images evidenced a compact structure for BracePaste in all environments sustaining the lower liquid absorption values. Fuji Ortho and Transbond present a fissure network allowing the liquid to carry out in-depth penetration of materials. SEM observations are in good agreement with the atomic force microscopy (AFM) results. The surface roughness decreases with the acidity increasing for BracePaste meanwhile it increases with the acidity for Fuji Ortho and Transbond. In conclusion: BracePaste is recommended for long-term orthodontic treatment for patients who regularly consume acidic beverages, Fuji Ortho is recommended for short-term orthodontic treatment for patients who regularly consume acidic beverages and Transbond is recommended for orthodontic treatment over an average time period for patients who do not regularly consume acidic beverages.

## 1. Introduction

Orthodontic problems often require bracket usage as a long-term treatment [1,2]. They are subjected to mastication forces which induce shearing stress into the bonding interface but they must also transmit the orthodontic forces to the teeth [3,4]. The factors involved in the successful transmission of forces during orthodontic treatment are the preparation of the surface of enamel, the characteristics of the bracket and the type of cement used for bonding [5]. Therefore, the properties and characteristics of the involved dental materials need to be considered and g an appropriate product for successful performance needs to be chosen.

Materials used for orthodontic bonding usually fail as a result of micro fissures infiltrated by oral bacteria, or complete bracket debonding [6]. The ideal cement should perform enough retention to provide resistance during normal masticator forces, to transmit orthodontic forces and to be easily removable without causing damages to the surface of the enamel [7]. The two materials frequently used for bonding are composite resins [8,9] and glass ionomers [10,11].Composite resins were introduced by Buonocore and are most frequently used for bonding brackets due to their bond strength that increases and doubles within 24 h [12]. The composites’ resins need micro-mechanical retentions of an acid-etched surface of the tooth with a primer-bonding agent to aid coupling of the two surfaces. The disadvantage of this method is the risk of enamel demineralization and the probability of enamel defects after bracket removal. That is the reason why composite resins were replaced with novel orthodontic cements based on fluoride-releasing materials, such as glass-ionomers cements [10,11].

Glass ionomers can release fluoride and prevent enamel demineralization, but they have a lower adhesion to the surface of enamel and they determine frequent debonding of the brackets, with a negative effect on orthodontic treatment. Glass ionomers modified with resins (RMGIC) were developed as a hybrid of glass ionomers and composite resins to ensure a better bond strength [13,14]. RMGIC have the advantages of composite resins and of glass ionomers: a better bond strength, fluoride-releasing properties and they can be used when it is difficult to dry the teeth (impacted canine, lower second molar, palatal/lingual surface, etc.). RMGIC preparation is based on an acid chemical reaction assisted by a light-activated additional polymerization of hydroxyethyl-methacrylate. Photo-polymerizable cements cure in approximately 30 s if exposed to a light source of the correct wavelength [15,16]. The acid-base reaction does not end when the photo polymerization process is complete, which means that the mechanical properties of the material are further improved. The difficulties of hand-mixing powder-liquid can provide us with errors, with a negative effect on the physical properties of the glass ionomers. This problem is solved by the use of encapsulation with fixed proportions by the manufacturer and mechanical mixing [15].

These material types are not new but the continuous challenge to improve their microstructure and mechanical properties is always of the great interest. The filler particles refinement and their embedding into the polymer matrix are constantly improved to release novel materials for dental use. Examples of orthodontic cement with newly improved formulation are: resin composites (e.g., BracePaste); resin-modified glass ionomer RMGIC (e.g., Fuji Ortho) and resin cement (e.g., Transbond).

A very important requirement of dental materials is to resist to the loads during mastication such as: shear, compression and flexing. The bonding layer between the enamel and metallic brackets is affected in a complex manner by compression, shear and flexing solicitations [12,14]. This complex behaviour can be quantified using standardized determination of the mechanical properties that include: diametral tensile strength, compressive strength, shear bond strength, and flexural strength. The results of these standard tests allow specialists to choose the proper orthodontic cement type to the specificity of the intended treatment. The orthodontic treatment time is one the most important parameters because the cement bonding must ensure the desired mechanical properties during this whole time period. A novel approach of the present research is to compare the behaviour of the three types of orthodontic adhesives in acidic conditions similar to the ones in the patient’s mouth.

The real conditions from the patient’s mouth represent an acidic environment caused by the low pH of food and drinks which may affect all three elements of the orthodontic treatment: enamel surface, bracket material and the bonding layer [17,18]. The bonding layer is very sensitive to the acid erosion only if it presents increased liquid absorbtion or a significant solubility in certain liquids such as acidic soft drinks [18,19]. The most popular acidic beverages among youngsters are Coca-Cola acidified with phosphoric acid and Red Bull containing a mixture of acids, with the most representative being citric acid [20]. If the microfissures occur in the adhesive layer they will generate microleackages [21] which may be decay accelerators when discussingthe acidic environment. Therefore, the orthodontic cements require the investigation of liquid absorbtion and solubility, microstructural aspects and surface topography in order to provide a completecharacterization. It is necessary for the dental practitioners to choose the proper cement for each orthodontic case.

The aim of present research is to realize a comparative investigation of mechanical properties such as: compressive strength, diametral tensile strength and flexural strength related to the liquid absorbtion, solubility and microscopic characterization of the following orthodontic cements: BracePaste, Fuji Ortho and Transbond. Shear bond strength compartative investigation for these materials in conditions similar to the patient’smouth is the challenge for the article.

The null hypothesis states that the mechanical properties are the same for all three of the investigated materials and that there are no microstructure, absorption and solubility differences between the exposure environments: artificial saliva, Coca-Cola and Red Bull.

## 2. Materials and Methods

### 2.1. Materials Description

The materials used in the present research are described in Table 1as follows: commercial product name, producer and the complete composition displayed on the product label. There is no other additional information regarding the used materials.

The complexity of the current paper requires various samples prepared from the materials described in Table 1 following the standard protocol of each investigation method. Therefore, the sample preparation is described for each experimental method in its subchapter.

### 2.2. Mechanical Properties

#### 2.2.1. Compressive Strength

The samples for compressive test were prepared in a Teflon/PTFE matrix with the shape of the disk being 0.6 mm thick, composed of two pieces with a cylindrical hole in the middle—with a diameter of 0.4 cm and a height of 0.6 cm. After polymerization, the resulting specimens were kept submerged in water for 24 h at a constant temperature of 37 °C.

Compressive strength was tested in a load range of 400–1100 N and the loading rate of 1 mm/min according to the American Dental Association (ADA) Standard No. 27. The value of compressive strength is the mean of 6 tests out of a total of 10. The specimens that exceeded the mean value by 15% were excluded. If more than 4 specimens fell outside the mean range, the testing for that series was repeated.

#### 2.2.2. Diametral Tensile Strength

The general technique for preparing and testing the specimens is similar to that used for compressive strength testing, with the exception of the specimen dimensions: 0.3 cm thickness and 0.6 cm diameter. The specimens are compressed along their diameter according to the American Dental Association (ADA) Standard No. 27. The application of loading force on the cylinder determines the stress on the vertical axial plane, as it is held between the two apparatus plates. Diametral tensile strength was tested at between 400–1100 N and the loading rate of 0.5 mm/min. The value of diametral tensile strength is the mean value of at least 6 determinations.

#### 2.2.3. Flexural Strength

The specimens used for flexural strength testing were polymerized in a Teflon/PTFE matrix in a parallelepiped shape—dimensions: 25 mm length, 2 mm width, 2 mm height. After polymerization, the specimens were submerged in distilled water for 24 h at the temperature of 37 °C.

To determine the flexural strength, the specimens were placed symmetrically on two supports with a 2 mm diameter, and the distance between the axes was l = 20 mm according to the ISO 4049/2000 Standard. The load F (N) that bends the specimen is applied centrally through a 2mm diameter cylinder. A3-point bending test was run with a loading rate of 0.5 mm/min. The value of flexural strength is the mean value of at least 6 determinations.

#### 2.2.4. Statistical Analysis

The results of the mechanical testing were analyzed using descriptive statistics (mean, median, standard deviation) and inferential statistics. One-way ANOVA of the 3 test groups was run for each group, and the significance level was set at α = 0.05. The statistical difference was analyzed using the Tukey test, with the use of Origin2019b Graphing and Analysis software.

### 2.3. Liquid Absorbtion and Samples Solubility

The composites are polymerized in a Teflon/PTFE matrix that produces specimens of the following dimensions: 15 ± 1 mm diameter and 1 mm thickness, to determine liquid absorption. The specimens were kept in a desiccator at 23 °C, before the initial weighing, until a constant weight was obtained (m1). Fifteen disk-shaped specimens were produced for each of the 3 sample groups, as per the ISO 4049/2000 standard. Five specimens of each material were placed in Group A and were immersed in a Cola-type beverage, another 5 were placed in Group B and were immersed in Red Bull, while the remaining 5 were the control sample and were immersed in artificial saliva. The specimens were desiccated and weighed immediately afterwards (m2), then after 2 h of recovery time in the desiccator (m3) at certain intervals (1 day, 2 days, 5 days, 6 days, 7 days, 14 days, 21 days). The liquid absorption (A) is calculated with Formula (1) and the samples solubility (S) is determined with Equation (2).

(1) $$A=100\times \frac{m2-m1}{m1}$$

(2) $$S=100\times \frac{m1-m3}{m1}$$

The same method of weighing the specimen disks immersed in the three liquids solutions used to determine absorption was used to determine the solubility of polymerizable dental composites, as per the ISO 4049/2000 standard.

### 2.4. Scanning Electron Microscopy SEM

The perfectly dried specimens used for the liquid absorption test were used for the scanning electron microscopy investigation. SEM images were obtained on the dried sample discs using a Hitachi SU8230 microscope, Hitachi Hi-Tec Corporation, Tokyo, Japan. The secondary electron images were obtained on the uncoated samples using an acceleration voltage of 30 kV and high vacuum chamber.

### 2.5. Atomic Force Microscopy AFM

Samples used for the atomic force microscopy are the dried specimens that were used for the liquid absorption. They were effectuated using a JEOL microscope (JSPM 4210, Tokyo, Japan). All the samples were investigated in tapping mode using NSC 15 cantilevers produced by MikroMasch, Bulgaria Headquarters, Sofia. The cantilever resonant frequency was 330 kHz, and the spring constant was 48 N/m. Three separate macroscopic areas were scanned for each sample at a scan size of 20 µm × 20 µm. The images were processed using the WinSPM 2.0 JEOL software (Tokyo, Japan) in accordance with standard procedures, presenting 2D topographic images, 3D images, and the Ra and Rq surface roughness parameters were measured. The 3D images are also called 3D profiles; they are a graphic representation of the depth profile of the enamel surface, closely related tothe measured values of surface roughness.

## 3. Results

### 3.1. Mechanical Properties

**Figure 1:**
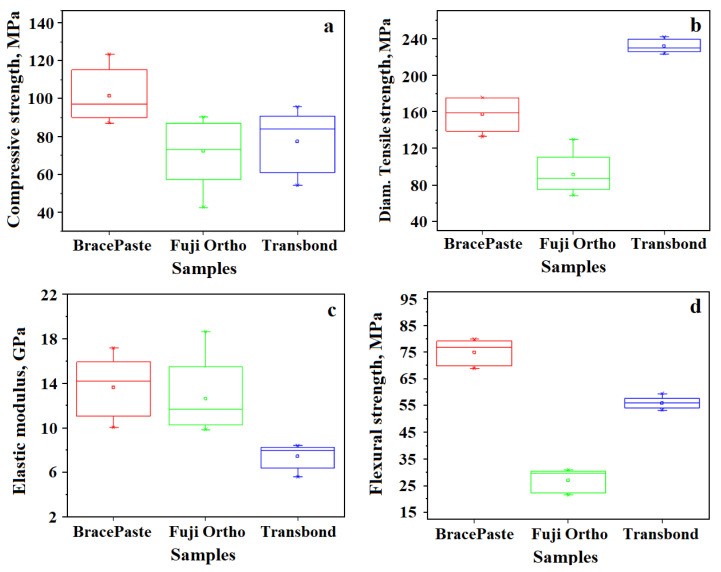
Mechanical properties variation for the initial cement samples with statistical analysis: (**a**) compressive strength, (**b**) diametraltensile strength, (**c**) elastic modulus and (**d**) flexural strength.

Compressive strength results are displayed in Figure 1a with the statistical analysis results. The best resistance was obtained by BracePaste around 100 MPa followed by Transbond around 80 MPa. The least resistant was the RGMIC sample, Fuji Ortho, having only 73 MPa. The differences in the compressive strength values are due to the particle-size distribution, where very fine powder particles insert themselves into larger ones, which lead to a decrease in the interstitial spaces between them. These inserted particles support the compressive stress, thus reducing the frequency of breakage.

Diametral tensile strength results are presented in Figure 1b and the statistical analysis results are displayed on the graph. The best compression strength was obtained for Transbond which was about 230 MPa. BracePaste and Fuji Ortho prove to be weaker under tensile load. The macroscopic examination of the samples after testing indicates a brittle fracture generated by the granular matter failure under axial effort. Therefore, the elastic modulus was calculated, Figure 1c. The results show that BracePaste and Fuji Ortho are more elastic than Transbond. This might be an important aspect concerning the material behavior under masticator forces.

Shear stress generated by the mastication induces flexural solicitation of the bonding layer. The flexural strength results are displayed in Figure 1d. The best result was obtained for BracePaste which was about 75 MPa and the lowest value was obtained by Fuji Ortho at about 27 MPa. This means that the higher concentration of filler determines the increased values of flexural strength.

### 3.2. Liquid Absorbtion

**Figure 2:**
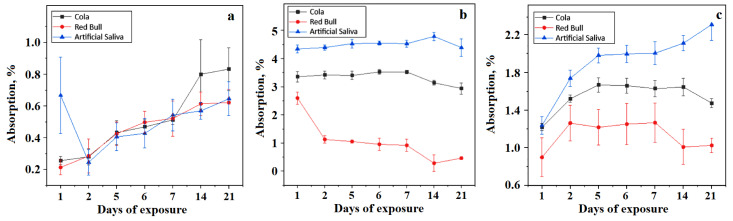
Absorption curveresults for the tested orthodontic cements: (**a**) BracePaste, (**b**) Fuji Ortho, and (**c**) Transbond.

Five polymerized specimens of each tested material type (BracePaste, Fuji Ortho and Transbond) were used for each immersion environment (Coca-Cola, Red Bull and artificial saliva). Absorption and solubility were tested at the following intervals: 24 h, 48 h, 5 days, 6 days, 7 days, 14 days, and 21 days. The study found that liquid absorption in the tested materials is dependent upon their chemical composition, the time factor, and the immersion medium, as shown in Figure 2. In the first 2 days, liquid absorption continues to significantly increase for all the tested materials, followed by a tendency to become stable until day 7, followed by another great increase in the majority of cases until the 21st day.

BracePaste absorbs more artificial saliva after 1 day of exposure and less Red Bull and the absorption is almost the same after 2 days of exposure. The situation evolves in a similar manner for all the exposure environments until 14 days, as shown in Figure 2a. After this point, we observed that the higher absorption occurs for Coca-Cola and the lower absorption occurs for the artificial saliva and Red Bull. This clearly indicates BracePaste sensibility to the phosphoric acid.

The results for Fuji Ortho, Figure 2b, and Transbond, Figure 2c, reveal that they absorbed more artificial saliva and less Red Bull. This behaviour may instead be explained by liquid viscosity, with Red Bull being more viscous than saliva than to its acidity.

Analyzing the absorption of the three types of materials in the same immersion medium, we can posit that all three materials have distinctly different behaviours, with the values of p much lower than 0.05. However, when immersed in Red Bull (*p* = 0.02), the glass ionomer material did not present any statistical differences when compared to the other two materials tested. Finally, the results show that BracePaste absorbs less liquid and Fuji Ortho presents the highest liquid absorption among the tested materials.

### 3.3. Solubility in Liquid Environment

**Figure 3:**
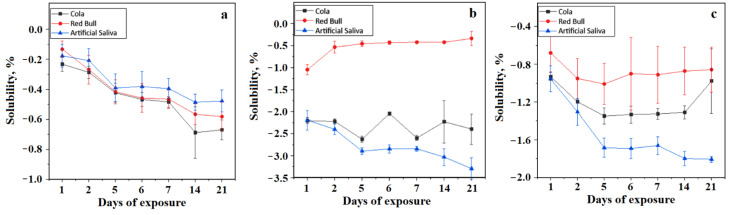
Solubility curve results for the tested orthodontic cements: (**a**) BracePaste, (**b**) Fuji Ortho, and (**c**) Transbond.

The bonding cement solubility is one of the most important physicochemical aspects regarding the integrity of the bracket adhesion on the teeth. If the adhesive is dissolved by the acid in food and drinks, bonding weakening takes place and this even affects the adhesion dissolution over time [22,23]. The percentage solubility is determined by samples weight and using Equation (2). The obtained values were centralized in Figure 3. BracePaste is more susceptible to being soluble in artificial saliva and less soluble in Coca-Cola over longer terms of exposure up to 14 and 24 days, as shown in Figure 3a. This is a very unusual aspect because the behavior in saliva must feature a strong stability not a predisposition to solubility [24,25,26], a fact which will be debated in the discussion section. However, the obtained values are below zero, and as a consequence, there is no dissolution danger to the patients during normal eating.

Fuji Ortho and Transbond present a similar behavior of being more susceptible to being dissolved in Red Bull and being less soluble in artificial saliva, as shown in Figure 3b,c. There is strong evidence that exposure to an acidic environment may facilitate local dissolution of the specific micro-structural components of these orthodontic cements.

By analyzing the solubility of the three types of materials in the same immersion medium, we can posit that all three materials have distinctly different behaviours when immersed in Coca-Cola (*p* = 1.99377 × 10^−12 2^); there are significant statistical differences (*p* = 2.52524 × 10^−4^) when they are immersed in Red Bull, without any differences between BracePaste and glass ionomer; there are significant statistical differences when they are immersed in artificial saliva (*p* = 3.30099 × 10^−11^) for all three of the tested materials.

### 3.4. Scanning Electron Microscopy SEM

SEM images were taken at average magnification to observe all the morphological details regarding the samples’ morphology. The interaction with the exposure environment is followed.

**Figure 4:**
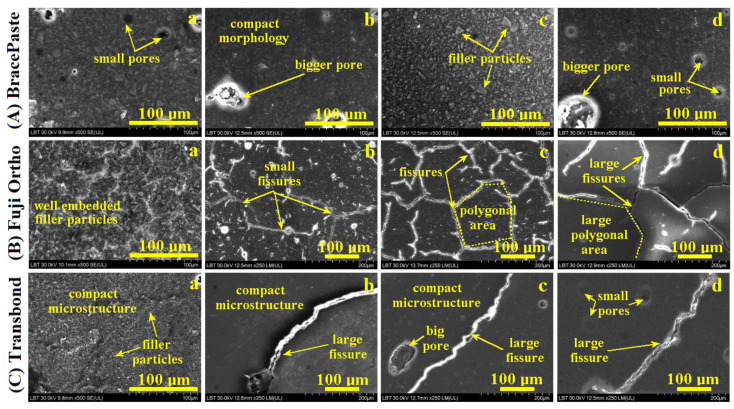
SEM images of the investigated samples: BracePaste (**A**); Fuji Ortho (**B**) and Transbond (**C**) exposed at different liquid environment: (**a**) initial—unexposed, (**b**) artificial saliva, (**c**) Coca-Cola and (**d**) Red Bull.

The initial BracePaste sample presents a relatively uniform and compact morphology due to the optimal cohesion between the polymer and mineral filler particles. Unfortunately, several pores occur: a few of them are bigger with rounded shapes and diameters of about 30 to 75 μm and several smaller pores with dendritic shapes are present, which thus means a more detailed topographic investigation is required, Figure 4A(a). The exposure to artificial saliva ensures a proper wetting of the sample surface (similar to the mouth conditions), a fact which improves the microstructure quality, Figure 4A(b). Exposure to an acidic environment reveals a good preservation of the samples’ microstructure, with only the outermost unevenness being eroded, Figure 4A(c,d).

Fuji Ortho RMGIC cement presents a typical microstructure with fluoro-aluminum-silicate glass particles very well dispersed in the polymer matrix. The silica nanoparticles are not visible, but their presence ensures the good consistence of the sample, Figure 4B(a). Proper cohesion between the filler particles and the polymer matrix is observed. The exposure to artificial saliva, Figure 4B(b), reveals a relative enhancement of the filler particle exposure to wet conditions in the outer most layers due to the liquid absorption. It generates a network of small fissures on the sample surface. Exposure to Coca-Cola generates more definite fissures propagated into a network which isolates polygonal areas with diagonal areas ranging from about 75 to 300 μm, Figure 4B(c). Exposure to Red Bull also generates thicker fissures propagated in a network, Figure 4B(d), that isolates polygonal areas with a greater diagonal area (e.g., 300–500 μm) than the one observed for Coca-Cola.

The initial Transbond sample, Figure 4C(a), revealed a compact and uniform microstructure which deals with refined filler particles such as amorphous silica. The exposure to the wet environment generates fewer strong fissures propagated over the samples surface, Figure 4C(b–d). A progressive corrugation of the outermost layer of the microstructure is observed especially for the exposure to Coca-Cola, Figure 4C(c), and to Red Bull, Figure 4C(d).

**Figure 5:**
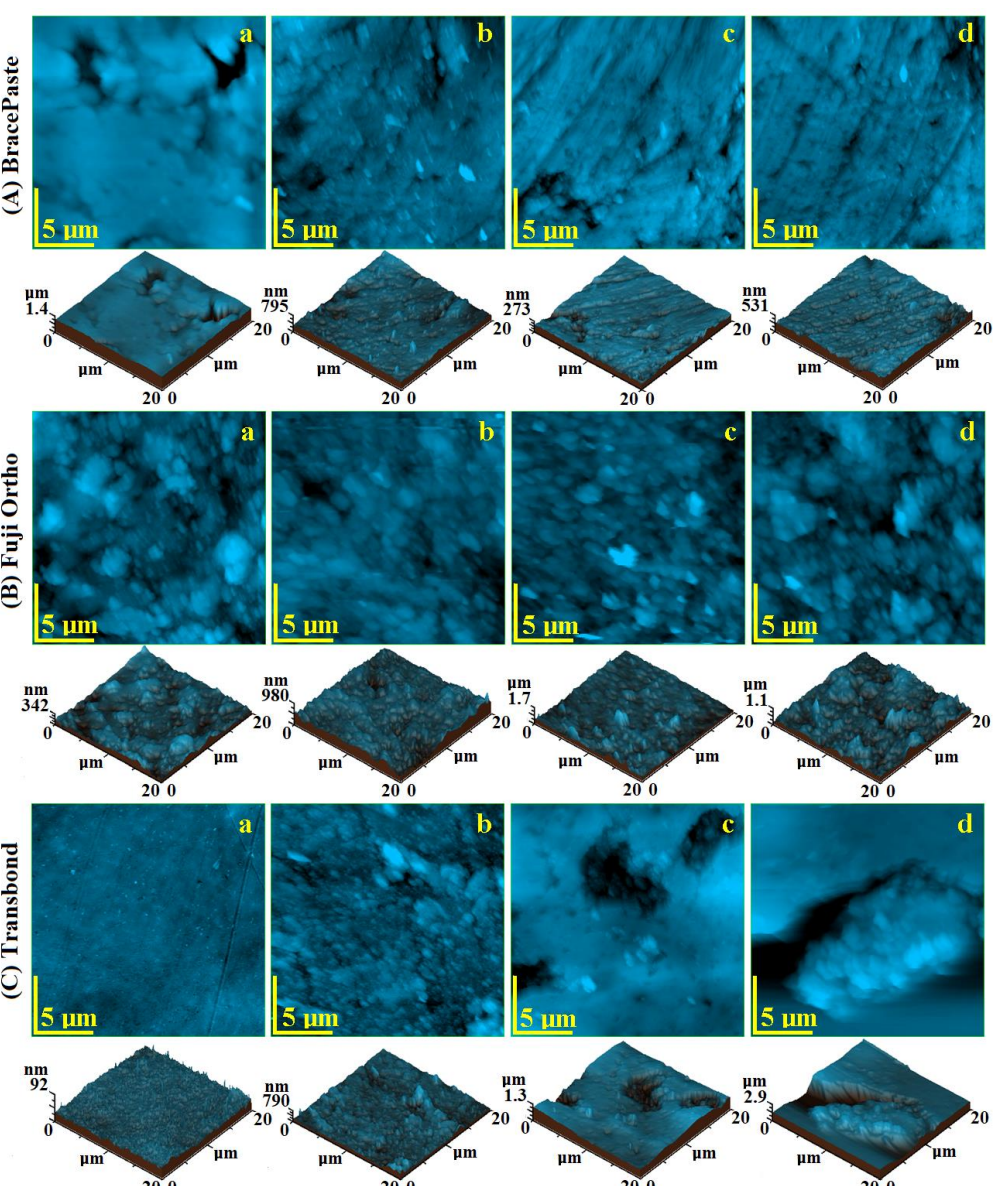
AFM images of the investigated samples: BracePaste (**A**); Fuji Ortho (**B**) and Transbond (**C**) exposed to different liquid environments: (**a**) initial—unexposed, (**b**) artificial saliva, (**c**) Coca-Cola and (**d**) Red Bull.

**Figure 6:**
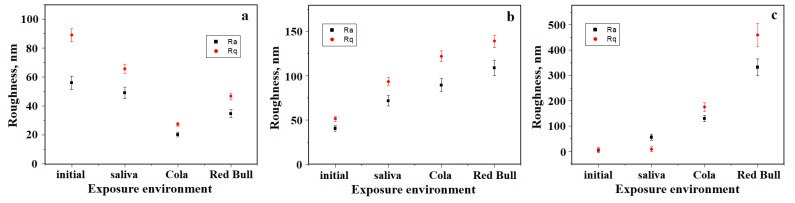
Roughness evolution after acidic environment exposure for the tested orthodontic cements: (**a**) BracePaste, (**b**) Fuji Ortho and (**c**) Transbond.

The surface topography of the initial BracePaste sample, Figure 5A(a), reveals a relatively uniform surface randomly punctured by the presence of some pores with a dendritic shape and dimensions ranging from 5 to 7 µm. They are more visible in the tridimensional profile given below the topographic image in Figure 5A(a) and they determine a relatively high value of the roughness. The exposure to artificial saliva tends to preserve the microstructural aspects, the dendritic pores’ presence is significantly reduced as observed in Figure 5A(b). This leads to the roughness decreasing. Exposure to an acidic environment contributes to an enhanced regularization of the topography with an attenuation of the pore depth as observed for Coca-Cola in the left lower corner in Figure 5A(c) and for Red Bull in the right upper corner of Figure 5A(d). This fact induces a strong decrease in the surface roughness, Figure 6a.

Initial Fuji Ortho presents a complex topography due to its various granular constituents. There are remarkable fluoro-aluminum-silicate glass particles with boulder shapes and diameters ranging from 3 to 5 µm and small submicron silica particles with diameters of 200–500 nm depending on their local arrangements, Figure 5B(a). The compactness between the polymer and mineral filler associated to a uniform surface ensures the relatively low value of the roughness. The exposure to the artificial saliva generally preserves the topographic features of the Fuji Ortho sample, with only some small alterations occurring due to the relative liquid sorption, Figure 5B(b). Therefore, the roughness increases slowly. The exposure to Coca-Cola presents an altered topography due to the acid erosion. Some of the fluoro-aluminum-silicate glass particles were delaminated from the polymer bonding and dislocated from the microstructure, Figure 5B(c), leading to the roughness increasing. The erosive aspect is more prone after Red Bull exposure, Figure 5B(d), which contributes to a severe increase in the roughness as observed in Figure 6b.

Transbond initial topography is presented in Figure 5C(a) and corresponds to a very uniform and compact surface. The filler particles are nanostructured and some of them form submicron clusters very well embedded into the polymer matrix. This fact determines a very low roughness value. The exposure to the artificial saliva facilitates liquid absorption on the superficial layers which causes a mild corrugation of the surface that unveils the filler particles, Figure 5C(b), which slowly increases the roughness value. Exposure to the phosphoric acid within Coca-Cola causes significant acid erosion which leads to the development of topographical depressions generated by filler particle loss, Figure 5C(c). Red Bull proves to be more erosive, Figure 5C(d), by generation of enlarged and deeper depressions on the sample surface. The acid effect causes a greater increase in the surface roughness as observed in Figure 6c.

Statistical analysis performed on the roughness measured for all the samples involved in the present research was not relevant due to the opposite behavior of BracePaste samples compared to Fuji Ortho and Transbond. This particular behavior is in strong connection with the observations on liquid absorption and microstructure local solubility.

## 4. Discussion

The materials used in dentistry must be durable and provide long-term adhesion, despite the hostile nature of the environment—there is permanent contact with saliva, a bacteria-rich fluid that contains multiple other organic and inorganic substances [24,25,26]. The interaction between saliva and the structural elements of the cements can cause the degradation or dissolution of superficial strata, the release of unbound or insufficiently bound components in the cement structure, or water absorption in the cement itself [27].

The mechanical properties of the materials included in this study depend on the following characteristics of the cements’ components (organic and inorganic): mechanical strength of the filling material, dispersed phase consistency, dispersion nanoparticle geometry, dispersed phase orientation, dispersed and continuous phase compositions and ratios, as well as the relationships between the two phases. Our previous study indicates that the refined filler particles are better dispersed onto the polymer matrix thus assuring a good cohesion of the microstructure [28,29], a fact which is in good agreement with the data in the literature [10,23]. There is a correlation between the physical–mechanical properties of the cements used for bonding and the mass and ratio of inorganic phase components as observed by AFM microscopy, which is in good agreement with the SEM observations. This indicates that the filler amount is an important factor but also the particles’ refinement and their proper dispersion into the fluid polymer before solidification plays a key role in the mechanical properties of the investigated materials. Therefore, BracePaste has the best compressive and flexural strength. High flexural strength is correlated with an increased elasticity modulus that ensures proper behavior during mastication. On the other hand, good compressive strength ensures good cohesion into the bonding layer, which is in good agreement with the literature [30]. Therefore, BracePaste is the best configuration for a strong adhesion between the enamel surface and brackets. BracePaste’s average diametral tensile strength does not affect long-term orthodontic treatment unless the brackets are accidentally pulled out. Transbond has the best diametral tensile strength, a good flexural strength, weaker compressive strength and low elasticity modulus. Its rigidity may affect the bonding layer behavior under long-term orthodontic treatment. However, the flexural strength of about 58 MPa ensures successful orthodontic treatment over an average time period, a fact which is in good agreement with the data in the literature [31].

On the other hand, Fuji Ortho presents low values of mechanical properties especially compressive and flexural strength because of the bigger filler particles (e.g., fluoro-aluminum-silicate glass) which are relatively brittle and tend to crack during the force load and the resulting fragments tend to further section the polymer matrix that acts as a failure promoter. However, the heterogeneous filler mixture observed in the Fuji Ortho RMGIC proves to be beneficial to achieving a good value of the elastic modulus, which is in good agreement with the requirements for a bracket adhesive [32,33]. Therefore, the first part of the null hypothesis regarding mechanical properties was rejected.

In the case of resin-based cements, liquid absorption can have beneficial effects and the possibility of lessening the residual polymerization contraction stress inside the organic matrix [16]. Results show that BracePaste has a greater liquid absorption in saliva [24,25,26] than in Red Bull, a fact that is related to the liquid viscosity and saliva being less viscous than energy drinks. Oberhofer et al. noticed this aspect in his study regarding the impact of energy drinks on young people and children [34]. The lower penetration of energy drinks among the filler particles proves to be protective against acid erosion propagation on the adhesion layer bulk. In the case of photo-polymerizable glass ionomer cements, some studies have shown that liquid absorption occurs for several months after the initial polymerization, which lessens their rigidity and strength [17]. Resin-modified ionomer cements undergo a rapid and marked expansion if immersed in an aqueous medium after hardening. This expansion compensates for the initial contraction, but may also induce fissures on the sample surface as observed in the SEM images. It has been demonstrated that RMGIC adsorbed more liquid than the composite resin [26] because of the hydroxyethyl methacrylate (HEMA) presence in the structure which is hydrophilic. Even if liquid absorption is characteristic of resin-based materials, the obtained results demonstrated that it has a negative effect on the mechanical properties, especially by decreasing flexural strength. The null hypothesis regarding liquid absorption and samples’ solubility was rejected.

The data in the literature mention the negative effect of acid erosion on the mechanical properties of the orthodontic cements’ adhesion between enamel and bracket. There are a few evidenced ways of erosion such as: enamel–cement interface decay, bracket–cement interface failure or cement layer internal failure [35,36,37]. The AFM observation proves that BracePaste presents a good behavior under acidic environments such as Coca-Cola and Red Bull because of the strong cohesion between the filler particles and the polymer matrix which ensures proper insulation of the mineral particles from contact with acids in the mentioned soft drinks. Fuji Ortho is significantly affected by acid erosion and Transbond is the most affected by acid erosion, a fact sustained by the surface roughness variation. This is due to the combination between the erosive action of the acid on the mineral filler particles and the fissures observed on their microstructures. We noticed the aggressive effect of phosphoric acid from Coca-Cola and citric acid in Red Bull in a previous study regarding CR and RMGIC materials used for inlays and crown adhesion [38].This corresponds to the data mentioned in the literature [39,40]. It is a matter of the filler particles’ distribution in the polymer matrix and their interaction with the exposure environment. The roughness decrease under acid exposure sustains the BracePaste resistance against in-depth penetration of the erosive agent and therefore ensures long-term protection of the bonding layer within the orthodontic treatment. The fissure and pores’ development under acid exposure evidenced for Fuji Ortho and Transbond causes the surface roughness to increase as a consequence of the erosive effect. The different behavior observed on the investigated materials under acidic conditions rejects the null hypothesis. Therefore, it is necessary that the dentist asks the patient what their diet habits are (e.g., if they regularly consume acidic beverages or not) prior to establishing the cement to be used for the orthodontic treatment.

## 5. Conclusions

The investigated orthodontic cements present a complex relationship between their mechanical properties, microstructures and their behavior in acidic condition. They allow dentists to choose the optimal bonding of the brackets considering both the length of time of the orthodontic treatment and the patient’s feeding preferences:

1. BracePaste presents the best combination of mechanical properties and erosion resistance in various acidic environments, with it being recommended for long-term orthodontic treatment especially when the patient regularly consumes acidic drinks such as Coca-Cola and Red Bull. Prolonged exposure to acid overtime will make the debonding procedure at the end of the orthodontic treatment easy.
2. Fuji Ortho RMGIC features the best handling associated with easy bracket procedures and presents low erosive resistance overtime. Therefore, it is easy to be applied at the beginning of treatment and it is easy to use in debonding at the end of the orthodontic treatment with minimal discomfort for the patient. Beside these important aspects, it is recommended only for short-term orthodontic treatment and is suitable for patients who regularly consume acidic beverages such as Coca-Cola and Red Bull.
3. Transbond presents the highest diametral tensile strength but its compressive and flexural strength are inferior to BracePaste, and it also presents poor erosive resistance in acidic environments. This adhesive may ensure good adhesion of the brackets for orthodontic treatment over an average period of time for patients who do not regularly consume acidic beverages.

## Figures and Tables

**Table 1:** Materials characteristics.

Product Name	Producer	Composition
BracePaste	American Orthodontics, Sheboygan, WI, USA	Methacrylic acid ester, activator, Ethoxylated Bisphenol A, Dimethacrylate, Tetramethylene Dimethacrylate, Diphenyl (2,4,6-trimethylbenzoyl) phosphine oxide.
Fuji Ortho LC	GC Company, Tokio, Japan	20% Polyacrilic acid Fluoro-aluminium-silicate glass Polyacrilic acid, HEMA, UDMA, silicon dioxide, distilled water, initiators, pigment.
Transbond Colour Change	3M Unitek, St.Paul, MN, USA	35%Phosphoric acid Primer- bis-GMA, TEGMA Adhesive paste- bis-GMA, TEGMA, Silane, treated quartz, amorphous silica, camphor quinone.

## Data Availability

The data presented in this study are available on request from the corresponding author.
